# The Challenges of Conducting Qualitative Research on “couples” in Abusive Intimate Partner Relationships Involving Substance Use

**DOI:** 10.1177/1049732320975722

**Published:** 2020-12-08

**Authors:** Beverly Love, Juliet Henderson, Amy Johnson, Danielle Stephens-Lewis, David Gadd, Polly Radcliffe, Elizabeth Gilchrist, Gail Gilchrist

**Affiliations:** 1 King’s College London, London, United Kingdom; 2 The University of Edinburgh, Edinbrugh, United Kingdom; 3 University of Gloucestershire, Cheltenham, United Kingdom; 4 The University of Manchester, Manchester, United Kingdom

**Keywords:** domestic, abuse, substance use, addiction, gender, qualitative, reflexivity, UK

## Abstract

Undertaking qualitative dyad or couple interviews involving intimate partner abuse and substance use presents considerable ethical, safeguarding, and theoretical challenges throughout the research process from recruitment to conducting interviews and analysis. These challenges and how they were managed are outlined using the experience from a qualitative study of 14 heterosexual “couples” that explored the complex interplay between intimate partner abuse and substance use. Managing these challenges for participants, their families, and researchers included the use of safeguarding protocols and procedures to manage risk and the provision of clinical support for experienced researchers. Researchers often felt drawn into the conflicts and complex dynamics of opposing accounts from the male and females’ relationship which could be emotionally and methodologically taxing. Researchers discussing their analysis and felt experiences with each other provided a reflexive space to manage emotions and stay close to the theoretical underpinnings.

## Introduction

Qualitative research where both the men and women in an intimate relationship are interviewed either together or separately has been used to explore shared experiences such as in relationships where illness has featured (Forbat & Henderson, 2003), health problems (Zarhin, 2018) and in intimate second relationships in later life (Eisikovits & Koren, 2010). Some authors refer to this as a dyadic approach, which focuses on the relationship as the main unit of analysis and aims to provide an “understanding of the relationship between two people” (Thompson & Walker, 1982, p. 889). Polak and Green (2016) have highlighted how the qualitative dyadic interview method has been interpreted differently within research. They have distinguished between contrasting data collection approaches (interviews conducted jointly or separately), analysis (multiple perspectives on shared experiences; shared perspectives on the same experience, Taylor & de Vocht, 2011), and the relationship between the dyad (linked pairs such as intimate couples or unrelated, such as a patient and their support worker, Caldwell, 2014; Morgan et al., 2013). The different approaches provide advantages and specific challenges in research where intimate partner abuse and substance use is involved. For example, having both perspectives on a shared experience such as an intimate relationship can provide an enriched understanding of the relationship dynamics.

While there is a long history of quantitative research involving couples in abusive intimate partner relationships (Straus et al., 1996), there are few studies based on qualitative interviews with both partners, either separately or together (Band-Winterstein & Eisikovits, 2009; Boonzaier, 2008; Hydén, 1994). In this article, we discuss the approach taken and the challenges faced when conducting separate interviews with men (in substance use treatment who reported having perpetrated intimate partner abuse) and their current or former female partners.

## Rationale for Using Qualitative “couple” Interviews to Understand Intimate Partner Abuse

Violence against women, of which intimate partner abuse is the most common form, has been deemed a significant public health concern by the World Health Organization (WHO; 2019). While there is a long history of quantitative dyad research using survey data to determine the prevalence of intimate partner abuse (including, psychological, controlling behaviors, financial control, physical or sexual abuse) conducted with couples in intimate relationships, it has been criticized for focusing on discrete events only and not being able to adequately capture patterns of abuse or violence (Allen, 2011; Dobash et al., 1998; Kimmel, 2002). “Family violence” researchers using the Conflicts Tactics Scale (CTS; Straus et al., 1996) in general population surveys of couples have argued that men and women report similar rates of violence perpetration and victimization. Such findings have led to a debate on whether there is *gender symmetry* in intimate partner abuse perpetration (i.e., whether women perpetrate intimate partner abuse to the same extent and with the same level of severity as men; Brown, 2012). However, the use of the CTS has been criticized as it measures events rather than the pattern and context of intimate partner abuse (Allen, 2011; Dobash et al., 1998; Kimmel, 2002). In addition, the CTS excludes intimate partner abuse by ex-partners (which tends to be more severe; Kimmel, 2002). Both household crime victimization survey research and feminist research have demonstrated that women experience abuse more frequently and more severely than their male counterparts, that their use of violence is predominantly in self-defense (DeKeseredy, 1997), and that the understanding of intimate partner abuse in terms of discrete acts ignores the context of aggression and controlling behavior by men to their current and former partners (Dobash & Dobash, 2004).

The authors argue that the use of violence to control one’s spouse is very different from the use of violence to protect oneself. Simply to ask who hit whom, and how often, thus risks missing such important contextual factors. Research has generally focused on either the victim or perpetrator’s perspective. However, prioritizing one account offers only a partial picture of the relationship and events. Interviewing both the man and his current or former partner separately to capture the accounts of both people involved provides a greater understanding of the dynamics of intimate partner abuse and can provide an enriched account of that relationship.

Band-Winterstein and Eisikovits (2009) interviewed older heterosexual couples separately to explore how intimate partner abuse unfolds over time. The couples’ separate perspectives on the violence in their relationship, elicited during interview, were considered the primary unit of analysis to provide context and depth to the findings. Care was taken not to assume one account was more accurate than the other, but researchers used their “judgment to establish the level of relative violence” (Band-Winterstein & Eisikovits, 2009, p. 167). The latter point emphasizes the considerable skill involved in the interpretation of dyad interview data concerning domestic abuse. Analysis required sensitivity to the misuse of power entailed in domestic abuse and that perpetrators may be motivated to present themselves in a positive light (Presser, 2004). Using a narrative approach, Boonzaier (2008) conducted separate interviews with heterosexual couples in South Africa where men had perpetrated domestic violence. Interviewing both parties in the relationship was considered to provide a less “one sided” account and in particular to understand men’s perspectives of perpetration in addition to the women’s in the same relationship. In this study, women were recruited after their partners had been interviewed which was considered safer as female victims often keep their involvement in such research secret to avoid conflict with their partners. Hydén (1994) interviewed 20 Swedish heterosexual couples to understand the narratives of marital violence. Interviews were conducted separately by different researchers as well as conjointly. Interviewing both men and women provided a “well-formed story” of lower level violence that the author argued is often not captured in police records. Key findings included men abdicating responsibility for their actions and that when marriage was of “great significance” to couples, both were more likely to “neutralise” or downplay acts of violence.

## The Complex Interplay of Substance Use in Intimate Partner Abusive Relationships—Enriching the Data by Interviewing Both Individuals in a Current or Former Relationship

Research shows that substance use is associated with higher rates of intimate partner abuse perpetration, with dependent use a stronger correlate of intimate partner abuse perpetration for males than substance use alone (Cafferky et al., 2018). There remains a need to conduct qualitative research to better understand intimate partner abuse within the context of substance dependency (Cafferky et al., 2018) to inform tailored interventions for this group (Gilchrist & Hegarty, 2017). Despite studies exploring intimate partner abuse within the context of substance use, no previous studies had involved qualitative dyad interviews where both partners were interviewed in-depth about their relationships (Gilchrist et al., 2019).

Our study responded to this gap and the need to understand the range of influences and interactions between substance use and intimate partner abuse, by conducting separate interviews with 14 men and their current or former female partner. Our research did not seek one “truth” but sought to interrogate the different perspectives regarding what happened, while being mindful not to excuse the perpetrator or blame the victim. The individual interviews highlighted the range of behaviors and experiences of both parties and allowed detailed analysis of abusive behavior in the context of substance use. This provided a rare opportunity to develop a more multifaceted understanding of what some victims regarded as a pattern of intentionally controlling and intimidating behavior could be cast as a singular and unfortunate incident by some perpetrators.

While our study findings identified some key benefits of interviewing both the man and his current of former partner (such as discrepancies in accounts) where intimate partner abuse and substance use dependency were involved, it also presented considerable ethical and safeguarding challenges at every stage of the research process from recruitment to interviewing and data interpretation. Managing risk for participants, their families, and researchers was a priority. This article provides guidance for researchers’ wishing to undertake qualitative research with couples from complex populations by illustrating the considerable skills involved, as well as the challenges and how risks were managed.

## Overview of the Research Study

The interview data comprised the narratives of male intimate partner abuse perpetrators who use substances (those with problematic use such as dependency) and their female current or former partners’ narratives of what contributed to intimate partner abuse at the same time exploring the role of substance use in the perpetration of intimate partner abuse in intimate relationships (Gadd et al., 2019; Radcliffe et al., 2019). Males attending substance use treatment who disclosed ever having perpetrated intimate partner abuse were recruited from six community-based substance use treatment settings in London and the West Midlands. Men provided researchers with the contact details for their current or former female partners, to allow researchers to invite them to be interviewed after the man had been interviewed. An adapted form of the Free Association Narrative Interview Method (Hollway & Jefferson, 2008), which incorporates reflective techniques, was used to elicit participants’ stories of their relationships, intimate partner abuse, and substance use. Analysis included establishing a timeline of events and noting similarities and differences in accounts. These analyses were synthesized first into individual and then couple case studies (“pen portraits”), to provide a fuller picture of the relationship and the complex interplay of intimate partner abuse and substance use. Further analysis combining narrative and thematic approaches (Floersch et al., 2010) using abductive and deductive techniques was used to develop codes relating to the involvement of substance use in abusive relationships (Braun & Clarke, 2006). These codes were further refined using narrative criminological approaches focusing on identity construction among violent offenders and their rational for their behaviors (Brookman, 2014; Presser, 2004, 2009). NVivo was used to manage and capture the analytical coding process.

The research is part of a larger funded program by the National Institute for Health Research (Advancing theory and treatment approaches for males in substance use treatment who perpetrate intimate partner violence, P-PG-1214-20009), which aimed to develop and test an evidence-informed intervention to reduce intimate partner abuse perpetrated by men in substance use treatment. The research received approval from the London Stanmore National Health Service (NHS) Research Ethics Committee (reference: 17/LO/0395).

## Ethical, Legal, and Safeguarding Challenges of Recruiting Both Individuals in a Current or Former Relationship

Recruitment of ‘couples’ (current or former partners) to a study concerning the sensitive topic of intimate partner abuse and among people who are using substances represents considerable challenges (Fraga, 2016; Neale et al., 2005; Rhodes, 2000). Seventy men, identified by keyworkers at treatment services, were screened by researchers for lifetime perpetration of intimate partner abuse, including physical, emotional, and/or sexual abuse, using a brief screening questionnaire developed for the purpose of this study, including questions modified from the WHO Multi-country Study on Women’s Health and Domestic Violence (Garcia-Moreno et al., 2005). Forty-seven men were eligible to take part and 37 were interviewed (Gadd et al., 2019; Radcliffe et al., 2019). Researchers received the contact details of 32 current or former female partners from 27 men recruited to the study following screening (73%). It was particularly difficult to recruit men’s current or former female partners who had experienced intimate partner abuse to the study. Many of the men were no longer in contact with their former partners. Only 17 of the 32 current or former female partners were contactable. Fourteen female current or former partners agreed to participate and were interviewed.

Recruiting both male perpetrators of intimate partner abuse and their female current or former partners presented distinct safeguarding and legal challenges. Failure to recruit and attrition in the recruitment process occurred for a number of reasons. Staff at one substance use treatment service advised against interviewing a man due to his severe mental health problems. The staff feared interviewing him may have presented further safeguarding issues for his partner and for the researchers. Men with “orders” preventing them or anyone from contacting their current or former partner on their behalf were not eligible to take part in the study (“orders” which are used by courts in England and Wales to protect victims and to prevent further harm, Crown Prosecution Service, 2019), as participation would have resulted in violation of the order. Researchers contacting partners in these cases may have provided an excuse for men to contact their (former)partner. Two men were excluded on these grounds.

Three females lived abroad so were not contacted as it was not possible to provide them with support or for safeguarding measures to be taken. Three women declined to be interviewed, one stating she did not want to recall the painful details of her past relationship. Staff at the treatment services advised against interviewing a further two women because they had recently relapsed into drug use. The impact on fragile recovery of disclosing sensitive and traumatic events was therefore considered a possible risk for participants. The risk of retraumatizing people and the possibility of this leading to relapse has been previously detailed (Bernstein, 2000; Love et al., 2019).

Safeguarding vulnerable participants and a duty of care for those who have substance use problems thus took precedence over recruitment. Some of these recruitment difficulties were anticipated and appropriate steps taken (including recruiting from several treatment services to provide a wider pool of potential participants and providing support to female victims/survivors via women’s independent safety services). However, it should be recognized that recruiting a larger number of men and their current or former partners (which may be required in some studies) may pose a considerable challenge among this group.

## Bearing the Individual’s Accounts of the Relationship in Mind—The Interview Schedule

There are several ways to construct an interview schedule for a study that aims to interview current or former partners in a relationship, depending on whether couples are interviewed together or separately (Eisikovits & Koren, 2010). A decision was made to interview the current or former partners separately in this study to avoid putting the female at risk of further abuse. There was also a concern that men may have sought to control the interview process (Ellsberg & Heise, 2002). While interviewing the participants separately reduced the risk of further abuse for female participants, it also meant that male and female current or former partners sometimes referred to separate events or aspects of their relationship.

Questions were broadly similar for both the men and women participants, both interview schedules enquired about abuse in the relationship and the involvement of substance use. However, the topic guides for the male and female participants differed in how the questions on intimate partner abuse were posed and some of the sequencing of the questions. While men had been recruited from substance use services and were known to use substances, it was not assumed that women used substances. Men were asked about the story of their substance use before they were asked about their relationship, whereas women were asked first about the story of their relationship. In two cases, men provided information about their former partner’s substance use that women did not disclose in the course of the interviews; perhaps these women did not feel comfortable disclosing their drug use in an interview with a female researcher who may be perceived as not sharing their experiences (Lee & Boeri, 2017). However, we could not rule out the possibility that these women did not use drugs and that men might have been seeking to undermine their current or former partner’s reputation, thus discrediting their accounts.

The interview questions for both the male and female interviews were phrased in the manner of “Can you tell me the story of what happened?” The interview schedules included questions such as the following:

I wondered if you could tell me the story of that relationship? How did you meet, how did the relationship start? How did drugs/drink impact upon the relationship? Can you tell me the story of a particular example of the abuse—what happened leading up to it/during/after? What about the most recent time (or most serious?) your partner was abusive? (Asked to females). What about the most recent time (or most serious?) there was abuse with your partner? (Asked to males).

The construction of the interview questions can provide further opportunities to explore where accounts were similar or discrepant. The responsive interviewing techniques deployed can also help to illicit rich detail (Hollway & Jefferson, 2008).

## Limits to Confidentiality

For safety reasons, limitations to confidentiality were explained to participants. These included that any disclosure of significant risk of current or future harm to self or others such as current/former partners or children would be disclosed to the substance use treatment staff and where required, to the relevant authorities. Where disclosures of current or future intention to harm were made, risks were assessed by the substance use treatment service in accordance with their safeguarding procedure. In line with Jewkes et al. (2012) research on sexual violence, participants were “advised not to describe incidents that were not known to the authorities in such detail as to enable victim identification or enable the incident described to be specifically identified” (p. 8). Furthermore, the study protocol adhered to the recommendation that “researchers should continuously be mindful of the need to avoid particular types of incriminating disclosure and should warn the research participant whenever he or she may be providing too much detail” (Jewkes et al., 2012, p. 8). Confidentiality had to be breached on one occasion where there was an assessed risk of harm to staff in the services, researchers, and the man’s former partner.

## Safeguarding the Female Participant and Their Families—The Use of Women’s Support Services, Clinical Support, and Rigorous Protocols

Different researchers conducted interviews with the man and woman in each couple and interviewed them on separate occasions. Researchers did not share information about specific events described by male interviewees with the researcher conducting the female interviews until both interviews had been completed, unless it was considered to place the researcher at risk. This approach ensured researchers did not inadvertently reveal information disclosed during interviews to the other partner, which could lead to further conflict or safeguarding risks. Partners were not permitted to attend the interview. This was to minimize the risk of men using information disclosed in interviews against women and provoking further conflict (Ellsberg & Heise, 2002). All men were interviewed in a private room in a substance use treatment service. Women were interviewed mostly in substance use treatment services but interviews also took place in their homes or other locations. One female was interviewed at a children’s center due to child care responsibilities and safeguarding concerns. Her former partner had disclosed a history of extreme violence toward her and had a number of previous restraining orders. To safeguard researchers, where women were interviewed in a home, two researchers were present.

As set out in the study protocol, when making initial phone calls to invite the women to take part in the study, scripts were used to ensure consistency and safety. Researchers ensured they did not leave detailed messages with family members or on answer machines about the nature of the study. If it was not convenient to talk, women were asked for a convenient time to call back.

As recommended by the WHO (2001) guidelines, both men and women interviewed were provided with details of support services including specialist domestic violence services and psychological wellbeing services. At the end of the interview, researchers asked participants how they were feeling and (for the men) if they required further support from staff at the substance use treatment service for any issues raised during the interview. This was important as some of the participants were still in relationships with each other and living together. Research with survivors of domestic violence and abusive relationships has found that some participants experience taking part in research as supportive (Campbell et al., 2010). In other studies, participants also reported the therapeutic benefits of being involved in research concerning conflict in relationships (Gilchrist et al., 2017 [personal communication from the English cohort of the study]; Owen et al., 2006). These benefits were commented upon in this study, for example, one female commented on the positive effect of being listened to and the opportunity to talk about her violent relationship.

To minimize risk, men were not informed by the researchers if their current or former partner had opted to take part in the study because male participants, on reflection, might not want their partners to take part (Ellsberg & Heise, 2002), although couples still in relationships might disclose their involvement to each other. This was the case for two men who actively tried to prevent or control the interview process. In one case, where substance use treatment staff highlighted recent suspected intimate partner abuse in which police and social services was known to have intervened, an interview with a female had to be conducted at the substance use treatment service rather than at the home where she lived with the male participant. In this case, attempts to arrange and conduct the interview with the female at the service was thwarted on several occasions when the male partner insisted on accompanying her and staying with her at all times. The clinical lead for the study considered that although this woman’s story remained unheard, to continue to attempt to interview the female participant placed her at risk of further violence. Other studies have reported that perpetrators had actively prevented their partners from disclosing the abuse to police, and victims were reluctant to report due to fear of repercussions (Wolf et al., 2003).

Where couples lived together, it was more difficult for those women who might want to keep the content of their interview secret from their partner to do so. For example, one male participant wanted to know exactly what questions his partner would be asked and waited at the services in anticipation of her arrival for interview. He was heard telling his partner that he would be sitting outside the interview room until it ended. The researchers believed this was a warning for her not to disclose anything that might implicate him. The interview was short (20 minutes), and the participant only spoke about her partner in the most positive terms. The man, after the interview, had disclosed to the researcher of violence in his past relationships and substance use treatment service staff were aware of his violence in previous relationships. In both of these examples, the researchers perceived that controlling behavior by the men toward their partners was being played out in front of them.

## Researchers’ Safety and Felt Experiences of Conducting the Interviews

### Researcher’s Safety

In isolated incidents, despite comprehensive safety protocols and safeguarding measures, researchers were exposed to a level of risk to their personal safety. In one case, a male participant showed the interviewer a knife that he reported carrying to protect himself on the streets. As per the study protocol, the researcher informed a member of staff, and the knife was confiscated from the participant without incident. As a precaution, risk to his current partner was also assessed by the clinical lead of the study, but no further action was deemed necessary. In another interview, a male participant became agitated during the interview when asked about his restraining order; he also belittled one of the female interviewers by referring to her nationality in a derogatory manner. The interviewer decided not to probe further on this topic. However, unbeknown to the researchers at the time, he had uttered threats which were only picked up during the transcription. In a third case, despite two researchers attending an interview in one woman’s home, the interview was curtailed as researchers were concerned when the woman disclosed that her former partner (who was participating in the study) had been stalking her home and still had access to the building. These incidents provide further evidence for the need for researchers to be experienced and well trained (Ellsberg & Heise, 2002; Neale et al., 2005). When researchers conducted interviews out of the substance use treatment service, they first checked the security of the interview venue and travel and parking options for safety. When interviews were conducted in services, if researchers felt threatened by a participant, they were encouraged to ask a member of the substance use treatment service to accompany them to their car or transport option. For all interviews conducted in nonclinical settings, researchers checked in and out by telephone with an office-based member of the research team. An emergency code word was agreed should urgent help be required without alerting the participant. Strategies for ending interviews early in a safe and respectful manner were also deployed so as not to inflame situations (Trevillion et al., 2016). Other pragmatic safeguarding measures included conducting interviews in a living room rather than in a kitchen (where there is access to potential weapons such as knives) and sitting near to an exit should the researcher be required to leave quickly.

### Researchers’ Felt Experiences of Interviewing

While having protocols in place to safeguard and protect researchers (as well as training in de-escalation, breakaway/self-defense techniques, and training in domestic violence awareness), it has to be acknowledged that researchers bring with them different experiences, beliefs, and emotional reactions to the research encounter. As such, decisions and emotional reactions are made in the field and afterwards will vary. Dickson-Swift et al. (2009) acknowledged in their research the different impacts undertaking qualitative research involving sensitive and difficult accounts can have on researchers. Ellsberg and Heise (2002) have recognized the emotional impact that undertaking such work can have on researchers.

In addition to the need to protect the research participants, researchers have acknowledged the risks posed to them when conducting research on sensitive issues such as the perpetration and victimization of intimate partner abuse. Gilbert (2001) asserts that experiencing emotions is part of the process in a qualitative researcher’s role and being empathetic is an essential skill (Liamputtong & Ezzy, 2005) but can also affect researchers negatively (Dickson-Swift et al., 2009). As noted as common in domestic abuse research (Ellsberg & Heise, 2002; Fraga, 2016), researchers in this study also reported difficulties (including fear, anxiety, and nightmares) after listening to stories, particularly when brutal descriptions of violence had been described. These difficulties may have been more acute because female partners were also included in the research. It was therefore sometimes difficult not to be drawn into their relationships and to feel affected by their struggles, and the violence and abuse they had experienced. Other researchers have discussed vicarious trauma after listening to participants’ traumatic and unsettling accounts (Love et al., 2019; Sammut Scerri et al., 2012; Van der Merwe & Hunt, 2019). In this study, researchers were offered regular (group and individual) clinical supervision by a clinical psychologist to process the emotions they experienced from hearing these stories. Researchers made (anonymized) notes on their impressions and feelings about interviews they could bring to these sessions. It was also important that team members supported each other, in weekly catch-ups and debriefing conversations (including with their line managers). This provided researchers the opportunity to share how the interview experience had made them feel and allowed breaks from the interviewing process, as recommended by the WHO (2001) guidelines. This was especially important as recruitment took place in busy waiting areas of substance use services where patients’ behavior could be unpredictable and erratic. This added to the “emotional labour” expended during the research process (Dickson-Swift et al., 2009; Hochschild, 1983).

### “He said, she said”—Valuing Both Partners’ Accounts: Critical Reflective Lens and Theoretical Stance

To compare narratives in the analysis and to provide a synthesis of male and female (current or former partner) accounts, timelines for each participant were first created to map out the chronology of events. Case studies in the style of “pen portraits” were then written for each participant aiming to highlight “apparent contradictions, avoidances and implausible claims” (Gadd et al., 2019, p. 1040), before combining these to provide an overall picture of the relationship. We were thus able to see where accounts were similar and where they diverged and were discrepant, to understand how women and men might view and understand their relationship differently in relation to the substance use and intimate partner abuse. Further narrative and thematic analyses were conducted to help refine codes (Braun & Clarke, 2006; Floersch et al., 2010) with a focus on how participants constructed their identities (Brookman, 2014; Presser, 2004, 2009).

The construction of case studies revealed notable differences in men and women’s narratives and explanations for intimate partner abuse. While male partners tended to describe intimate partner abuse as situationally specific and isolated incidents, where substances had provided the context within which the violence had occurred, women were more likely to refer to the lasting impact, emotional and psychological toll of violence victimization. While some of the women reported substance use to be involved, they also referred to other factors which provided the context to the violence including mental health and prior childhood adversity (Radcliffe et al., 2019). Most importantly these findings were used to inform the development of an integrated therapeutic intervention for male perpetrators of intimate partner abuse who used substances (Gilchrist et al., 2020).

However, there was not always agreement in interpretations among researchers, highlighting the complexity of data which include accounts from both sides of a relationship alluding to complex dynamics, abuse, and substance use. For example, when conducting the analysis of one particular couple’s data, the researchers involved in interviewing the man and woman disagreed about the overall interpretation of the relationship. The researcher (A) who interviewed the male considered that he had been the victim of abuse by the female; however, the researcher (B) who interviewed the female believed this was not the case and that the male was minimizing and denying his behavior. After reading each other’s participants’ accounts, their interpretations remained, both researchers identifying most closely with their own interviewees. The transcripts were then studied by the entire research team and discussed over several hours. The discussion and further researcher’s reflections revealed that researcher A had felt sympathy for the man’s adverse and violent childhood, whereas researcher B had been moved by the female participant’s distress when recounting the violence as well as her timid demeanor. Both researchers felt protective of their participants’ stories, demonstrating that not only “emotional labour” (Dickson-Swift et al., 2009; Hochschild, 1983) but also emotional investment is expended during the research process in the participants. These insights enabled the analysis to move forward, as a third researcher (author) developed an analysis that attempted to keep all these multiple identifications in mind, facilitating an interpretation that recognized the complexity of the motivations informing both accounts and the absences that generated (Gadd et al., 2019).

In a candid article, Garfield et al. (2010) highlight the value of discussing one’s own interpretations with other researchers to help reveal what aspects of participants’ narratives resonate with individual researchers and to identify the reflexive lens through which the analysis takes place. This reflective process can expose and reveal unconscious bias and allow further interpretations to take place. A reminder of the theoretical underpinnings of the analysis to value each account also helped to reorientate the analysis (Forbat & Henderson, 2003). Researchers’ interpretations of participants’ accounts are influenced by their own preconceptions and bias (Willig, 2013). For example, as domestic violence researchers, we are aware that men minimize and deny their behavior and can portray themselves as the victim (Dobash et al., 1998; Radcliffe et al., 2019). At the same time, we are aware that childhood adversity, including experiencing physical abuse as children and witnessing parental intimate partner abuse, may make perpetrating abuse more likely (Gilchrist et al., 2017). We also believe that with the right help and tailored support, some men can change their behavior (Langlands et al., 2009). Using an approach that made it permissible to entertain multiple interpretations of narrative data and the dynamics that facilitated its production proved invaluable at enabling the complexities of narrative data regarding the same events but from different perspective to be actively explored and often led to further discoveries in the data with regard to the nature of the anomalies and contradictions it contained. Bellas (1999) recognized how researchers can get drawn into participants’ lives, making it difficult to be detached and unemotional (Dickson-Swift et al., 2009). Forbat and Henderson (2003) have noted the difficulty of not taking sides in research with both couples in a relationship, which they term “imbalance.” Boonzaier (2008) used reflective awareness of her personal bias and preconceptions of victims and perpetrators to counter “imbalance” and avoid taking sides. Conducting and analyzing data for both sides of a current or former partnership about intimate partner violence thus poses emotional challenges for the researchers, and while empathy and connecting with the participant is essential in qualitative research, it can also present particular challenges in the research process. Using a more critical reflexive approach (Cuncliffe, 2016) and opportunities to discuss interpretations further were essential in this study.

Reading interview transcripts also presented the opportunity for further reflection and sometimes the need to reassess decisions made in the field about safeguarding participants. Reassessment included discussing with colleagues and the clinical lead if further action was required. For example, one female’s transcript included several references to her partner stalking her at home. However, on review, there was insufficient evidence to suggest she was in imminent danger. The researcher was advised to contact the participant to offer her support from women’s support services (in addition to support offered at the interview) and to check if there had been any further safeguarding issues.

### Ethical Considerations When Disseminating Findings

Studies have reported the ethical challenges of displaying the findings of each pair in a couple together in the same publication, where confidentiality might be compromised, should they recognize each other in the extracts presented (Boonzaier, 2008; Ummel & Achille, 2016). Careful consideration is required of how to present findings together. In our study, many of the couples’ narratives contained accounts of arguments, abuse, and violence. In our reporting, some details were changed or omitted such as the sex of children or locations. In addition, we sometimes truncated extracts to ensure that pairs would not recognize their stories (Boonzaier, 2008).

## Conclusion

Conducting qualitative research with current and former partners of the same relationship involving intimate partner abuse and substance use presents a unique set of challenges for researchers at every stage of the research process from recruitment to analysis and dissemination of findings. Recommendations for the field include ensuring safeguarding measures for both research participants and researchers are in place and assessed on a case-by-case basis. What might be considered safe for one participant might present as a risk to another, such as interviewing women at home or in a service. Having a protocol in place informed by clinical, safeguarding, and research experts across the relevant sectors was essential in this research where researchers encountered many ethical and safeguarding issues. The inclusion of clinical leads with expertise to offer advice as and when required to both researchers and staff alike, and liaising closely with the staff at substance use treatment services were further important measures to safeguard participants, their families, and researchers. Researchers interviewing and analyzing the data of both individuals in a “couple” can feel drawn into the conflicts and complex dynamics of opposing accounts from the male and females’ relationship which can be emotionally taxing. It is recommended that researchers have a reflective space and opportunities to discuss their analysis and felt experiences with each other to manage emotions and stay close to the theoretical underpinnings. The provision of clinical supervision was also key in helping to alleviate the “emotional labour” (Dickson-Swift et al., 2009; Hochschild, 1983) involved in listening to accounts of sensitive topics and working with a population where risks are more complicated by interviewing couples involved in intimate partner abuse and substance use. The merits of using a qualitative approach where both participants who are or have been involved in the intimate relationship provided a fuller understanding of both perspectives of how substance use intersected in intimate partner abusive relationships.
